# Ventral hernia repair in India: a Delphi consensus

**DOI:** 10.1007/s10029-024-03062-4

**Published:** 2024-05-09

**Authors:** P. Chowbey, R. Wadhawan, D. Subramanian, D. Bhandarkar, J. Gandhi, K. L. Kumari, M. Baijal, M. Khetan, M. S. Kathalagiri, P. Khandelwal, P. Lal, P. Dasgupta, P. Balachandran, S. Dave, S. J. Baig, V. Soni

**Affiliations:** 1 Max Institute of Laproscopic, Endoscopic and Bariatric Surgery, Max Hospital, Delhi, India; 2 MAX Institute of GI, Bariatric, Laparoscopic and Robotic Surgery, MAX Super Speciality Hospital, Dwarka, Delhi, 110075 India; 3 Department of General Surgery and Bariatric Surgery, MGM Healthcare, Chennai, India; 4 Department of General Surgery, Hinduja Hospital, Mumbai, India; 5 Department of General Surgery, KEM Hospital, Mumbai, India; 6 Department of Surgical Gastroenterology and Bariatric Surgery, Yashoda Hospitals, Hyderabad, India; 7 Institute of Minimal Access, Metabolic and Bariatric Surgery Centre, Sir Ganga Ram Hospital, New Delhi, India; 8 Department of Laparoscopic Surgery, Bariatric Surgery, General and Gastrointestinal Surgery, Sparsh Hospital, Bangalore, India; 9 Department of General and Laparoscopic Surgery, Aadicura Hospital, Vadodara, India; 10 Department of Surgery, Maulana Azad Medical College, New Delhi, India; 11 Department of Colorectal Surgery, Hernia and Abdominal Wall Reconstruction, Gem Hospital, Chennai, India; 12 Department of General, Gastrointestinal and Bariatric Surgery, Apollo Hospital, Chennai, India; 13 Department of Surgical Gastroenterology, Bariatric and Robotic Surgery, Ramkrishna CARE Hospital, Raipur, India; 14 Department of GI, Minimal Access and Bariatric Surgery, Bellevue Clinic, Kolkata, India

**Keywords:** Ventral hernia, Laparoscopic, Minimal access surgery, Intraperitoneal onlay mesh

## Abstract

**Purpose:**

While research on inguinal hernias is well-documented, ventral/incisional hernias still require investigation. In India, opinions on laparoscopic ventral hernia repair (LVHR) techniques are contested. The current consensus aims to standardize LVHR practice and identify gaps and unfulfilled demands that compromise patient safety and therapeutic outcomes.

**Methods:**

Using the modified Delphi technique, panel of 14 experts (general surgeons) came to a consensus. Two rounds of consensus were conducted online. An advisory board meeting was held for the third round, wherein survey results were discussed and the final statements were decided with supporting clinical evidence.

**Results:**

Experts recommended intraperitoneal onlay mesh (IPOM) plus/trans-abdominal retromuscular/extended totally extraperitoneal/mini- or less-open sublay operation/transabdominal preperitoneal/trans-abdominal partial extra-peritoneal/subcutaneous onlay laparoscopic approach/laparoscopic intracorporeal rectus aponeuroplasty as valid minimal access surgery (MAS) options for ventral hernia (VH). Intraperitoneal repair technique is the preferred MAS procedure for primary umbilical hernia < 4 cm without diastasis; incisional hernia in the presence of a vertical single midline incision; symptomatic hernia, BMI > 40 kg/m^2^, and defect up to 4 cm; and for MAS VH surgery with grade 3/4 American Society of Anaesthesiologists. IPOM plus is the preferred MAS procedure for midline incisional hernia of width < 4 cm in patients with a previous laparotomy. Extraperitoneal repair technique is the preferred MAS procedure for L3 hernia < 4 cm; midline hernias < 4 cm with diastasis; and M5 hernia.

**Conclusion:**

The consensus statements will help standardize LVHR practices, improve decision-making, and provide guidance on MAS in VHR in the Indian scenario.

## Introduction

A hernia is a weakness or bulge in which organs or tissues from the abdomen may become trapped, resulting in discomfort and symptoms like pain [1]. The prevalence of abdominal wall hernias is 1.7% for all ages and 4% for those over 45 years, making them a relatively common condition. Worldwide, an estimated 20 million patients with hernias are treated each year [2]. A ventral (abdominal wall) hernia is surgically repaired. The best surgical procedure is usually determined by a variety of factors, including the hernia’s size, past surgeries, its location, and the patient’s overall condition [1]. The following are the various hernia repair techniques: extended totally extraperitoneal (eTEP), total extraperitoneal (TEP), mini- or less-open sublay operation (MILOS)/endoscopic MILOS (eMILOS), stapler, transabdominal preperitoneal (TAPP), transabdominal retromuscular umbilical prosthetic (TARUP), subcutaneous onlay laparoscopic approach (TESLA, SCOLA), preaponeurotic endoscopic repair (REPA), laparoscopic intracorporeal rectus aponeuroplasty (LIRA), intraperitoneal onlay mesh (IPOM), and IPOM plus repair techniques [3]. There is currently insufficient data to determine which approach—open or laparoscopic—will result in a better primary VHR [4]. Currently, the most frequently used treatments for abdominal wall hernias are laparoscopic IPOM and open sublay mesh repair, according to the VHR guidelines that have been published recently [5].

The most popular minimally invasive procedure for VHR is laparoscopic IPOM. Moreover, specific patients at risk of wound complications or those with bigger hernia defects are recommended for laparoscopic treatment [4]. Although numerous studies have examined the results of both laparoscopic ventral hernia repair (LVHR) and open ventral hernia repair (OVHR), the best surgical technique, as decided by short- and long-term outcomes, is still debatable [6]. There is a lack of high-level evidence in support of different types of VHR techniques and a lack of consensus on laparoscopic VHR techniques in India. Thus, standardizing the practice of LVHR is the need of the hour. A panel of experts assembled (1) To support and enhance the decision-making process for laparoscopic VHR; (2), to drive a consensus and standardize the practice of laparoscopic VHR; and (3) to identify limitations and unmet needs that affect optimal safety and clinical outcomes.

## Methodology

### Selection of the panel

A panel of 14 experts (general surgeons) participated in the consensus rounds and development of the manuscript. Panel members were carefully selected based on their wide clinical expertise and knowledge in the field. A chair was identified among the panel members to drive the consensus process.

### Evidence review

A literature review was conducted using the PubMed database to find the pertinent articles published between January 2011 and April 2023. Keywords such as “ventral hernia,” “repair technique,” “guidelines,” “defect size,” “umbilical hernia,” “diastasis,” “epigastric hernia,” “M5 hernia,” “subxiphoid hernia,” “incisional hernia,” “vertical,” “midline,” “laparotomy,” “subcostal hernia,” “L3 hernia,” “bowel injuries,” “neurovascular,” “injuries,” “hematoma,” “vascular injuries,” “pain,” “IPOM plus,” “TAP block,” “adhesions,” “BMI,” “ventral hernia,” “grade 3/4 ASA,” “factors influencing,” “procedure,” and “algorithm” were used.

### Consensus process

The consensus was designed using the modified Delphi method. Before the physical meeting, two rounds of consensus gathering were conducted using a questionnaire that was distributed online to 14 experts. An expert consensus meeting was held to discuss the final statements and recommendations. The discussion started with an overview of the reviewed literature on a specific topic. In Round 3, voting was conducted during the advisory board meeting with 13 experts. Upon completion of Round 3, the final statements were decided based on agreement among the expert panel members. This manuscript presents the consensus statements and an algorithm that was developed based on the consensus achieved in the discussions.

Questions or statements that did not achieve consensus in Round 1 were removed and modified questions were presented in Round 2. Similarly, those that did not achieve consensus in Round 2 were modified and presented for discussion and voting in Round 3 during the advisory board meeting. During the meeting, survey results were discussed with supporting clinical evidence. The expert panelists summarized the round 3 results. The discussions were based on the preferred procedures considering the following:

1. Different types of hernias based on defect location and size
2. Potential for the occurrence of bowel, neurovascular, and hematomas/vascular injuries
3. Postoperative pain
4. Adhesion with coated mesh
5. Body mass index (BMI) of the patient

The level of consensus was categorized into high (≥ 75%), moderate (60–75%), and low (< 60%) as shown in Table 1.

**Table 1:** Definition of the level of consensus

Level of consensus	Definition
High	When ≥ 75% of participants agree/strongly agree or disagree/strongly disagree with a statement
Moderate	When 60–75% of participants agree/strongly agree or disagree/strongly disagree with a statement
Low	When < 60% of the participants agree/strongly agree or disagree/strongly disagree with a statement

## Results

### Use of minimal access surgery (MAS) in VHR

#### Valid options for MAS in VH

The repair of VH can be done using a variety of laparoscopic procedures [7]. LeBlanc and Booth first described laparoscopic IPOM, one of the earliest minimally invasive techniques for VHR, in 1990, which was later improvised into IPOM plus [8]. When compared to an open procedure, laparoscopic IPOM repair had fewer postoperative complications, mostly related to wounds. However, laparoscopic IPOM does have some common side effects, including seroma development, bulging, and inability to restore abdominal wall function. Laparoscopic IPOM with defect closure (laparoscopic IPOM plus) has been recommended for the treatment of VH to improve outcomes [7]. A revolutionary method for VHR is eTEP [9]. It is a cutting-edge strategy that entails creating bilateral retro-rectus spaces and linking them using a minimal access approach [10].A systematic review and meta-analysis on the comparison of eTEP and IPOM in VHR and incisional hernia repair concluded that the eTEP approach exhibits much lower acute postoperative pain and shorter hospital stay despite the longer operational duration. Additionally, there is no discernible difference in intraoperative or postoperative complications between the minimally invasive eTEP and IPOM [11]. Laparoscopic Trans-Abdominal Retromuscular (TARM) employing polypropylene mesh (PPM) was successful in treating small, medium, and some large hernias [12]. The MILOS concept, developed by Reinpold et al. [13] in 2009, 3 years after the creation of eTEP, permits the use of larger meshes by a minor skin incision and laparoscopic retro-rectus space dissection, as the 2016 revision, avoiding the implantation of an intraperitoneal mesh. When it comes to repairing VH, MILOS is just as effective as more conventional methods, but with significantly lesser postoperative problems, reoperations, and unexpected readmissions. It is verified that the method is simple to replicate and workable at a public hospital [13, 14]. vTAPP appears to be a safe and effective therapy for VHR, superior to or comparable to other minimally invasive methods in terms of perioperative characteristics and short-term outcomes [15].

*Experts’ opinion/consensus recommendation:* Based on the literature review, IPOM plus, TARM, eTEP, MILOS, TAPP, TAPE, SCOLA, and LIRA were presented to the experts to determine the suitable options for VHR. The experts agreed that all were valid options with a high 93% agreement.

**Consensus statement 1:** IPOM plus/TARM/eTEP/MILOS/TAPP/TAPE/SCOLA/LIRA are valid MAS options for VH.

#### Valid MAS options for hernia defect ≤ 8 cm

Suture and mesh are the two basic types of repairs for umbilical hernias. Small defects upto 2 cm can be repaired simply with primary sutures. According to a postal survey from Scotland, surgeons favored mesh repair for abnormalities larger than 2 cm, whereas they preferred suture and mesh repairs equally for defects smaller than 2 cm [16]. As per the guidelines of the Society of American Gastrointestinal and Endoscopic Surgeons (SAGES), when adopting a laparoscopic technique, the size of the hernia defect is crucial to take into account because larger defects typically make the treatment more challenging. Although not a complete contraindication, a recent recommendation from the Italian Consensus Conference advised caution for defects larger than 10 cm. On the contrary, the same group advised against using laparoscopic surgery for hernias with a defect size of less than 3 cm [17]. The guidelines [International Endohernia Society (IEHS)—Part 1], recommended that ventral and incisional hernias should ideally be treated laparoscopically if the defect is less than 10 cm in diameter [18].

*Experts’ opinion/consensus recommendation:* Based on the literature review, IPOM plus, TARM, eTEP, MILOS, TAPP, TAPE, SCOLA, and LIRA were taken under consideration as feasible surgical options for widths up to a maximum of 8 cm to enable tension-free closure. The experts agreed that all were feasible options with a moderate 71% agreement.

**Consensus statement 2:** IPOM plus/TARM/eTEP/MILOS/TAPP/TAPE/SCOLA/LIRA are usually feasible for detecting widths up to a maximum of 8 cm to enable tension-free closure.

### Midline hernias

#### Preferred MAS procedure for primary umbilical hernia < 4 cm without diastasis

Umbilical hernias are midline hernias that extend from 3 cm above to 3 cm below the umbilicus, according to the European Hernia Society’s classification for primary abdominal wall hernias [16]. Mesh reinforcement in open repairs of small umbilical hernias has consistently been shown to reduce recurrence rates. Few published retrospective studies found recurrence rates for suture repair to be 4–15%, whereas for mesh repair the rates were substantially lower at 0–5% [19–23]. A study by Kaufmann et al. [24] was conducted to investigate whether mesh repair was more effective than suture repair at preventing the recurrence of small umbilical hernias (diameter 1–4 cm). This study demonstrated the significant benefit of using mesh in the management of small umbilical hernias of 1–4 cm [24]. Additionally, a meta-analysis of randomized controlled trials (RCTs) revealed that open mesh repair of umbilical hernias was linked to a significantly decreased recurrence rate [odds ratio (OR) 0.22, 95% confidence interval (CI) 0.10–0.48; p = 0.0001] compared to suture repair [25]. As per the IEHS guidelines, mesh repair is recommended in all patients with an umbilical hernia of size 1–4 cm [26].

*Experts’ opinion/consensus recommendation:* For primary umbilical hernia of < 4 cm without diastasis, intraperitoneal (IP) and extraperitoneal (EP) were presented as options for MAS procedures to the experts. The experts agreed that IP is the preferred MAS procedure with a high 79% agreement.

**Consensus statement 3:** IP is the preferred MAS procedure for primary umbilical hernia < 4 cm without diastasis.

#### Preferred MAS procedure for umbilical hernia with diastasis < 4 cm

“Diastasis rectus is a condition in which the linea alba thins and the gap between the recti abnormally increases without a concurrent fascial defect” [27]. The operative management of a rectus diastasis and a concomitant umbilical or epigastric hernia presents a significant challenge [28]. According to reports, the recurrence rate was found to be higher in patients after small umbilical or epigastric hernia repair with a concomitant rectus diastasis compared to that in patients without a rectus diastasis [28]. Based on the limited information available, umbilical hernia combined with rectus diastasis can be repaired using open and endoscopic procedures. As the presence of a rectus diastasis seems to be associated with an increased risk of hernia recurrence, mesh augmentation of the hernia is recommended. It is optional to simultaneously correct the diastasis; this should be discussed with the patient [28]. When seeking to prevent potential issues linked to intra-abdominal mesh positioning, the laparoscopic TAPP technique for umbilical hernia and rectus diastasis may be a safe surgical choice [29]. Shinde et al. [27] examined the SCOLA with an added modification of the operating port, using spinal needles to restrict the amount of lateral dissection, and found that with little risks and postoperative morbidities, SCOLA is an effective surgery for treating umbilical/epigastric hernia with diastasis recti [27].

*Experts’ opinion/consensus recommendation:* The preferred MAS procedure options recommended to the experts for umbilical hernia with diastasis less than 4 cm were IP, EP, and endoscopic onlay procedures. IP achieved 21% consensus, endoscopic onlay achieved 29% consensus and EP achieved 50% consensus.

**Consensus statement 4:** The consensus was not achieved as the experts were unable to come to an agreement on the preferred MAS procedure for umbilical hernia with diastasis < 4 cm.

#### Valid MAS procedure of choice for a primary epigastric hernia of width < 4 cm

Epigastric hernias can be surgically repaired with a laparoscopic or open procedure using either simple suture repair or mesh reinforcement [30]. In clinical practice, the surgeon’s preferences, patient characteristics such as such as comorbidities, patient expectations, and hernia characteristics, like, defect size, hernia location, and reducibility, may all have an impact on the surgical approach for these hernias [30]. A few studies have been performed on the surgical treatment of primary epigastric hernias. The best available data suggests that mesh reinforcement in primary epigastric hernia repair may minimize recurrences and that laparoscopic surgery improves postoperative pain [30]. As per the algorithm for the repair of symptomatic umbilical and epigastric hernia, an open approach with a preperitoneal flat mesh is advised for medium-sized (greater than 1 cm in length and up to 4 cm) umbilical or epigastric hernias. Patients with multiple defects or those who are at a high risk of wound complications may be candidates for this laparoscopic technique [28].

*Experts’ opinion/consensus recommendation:* For a primary epigastric hernia with a width of < 4 cm, IP, EP, and endoscopic onlay were the recommended MAS procedures. With a moderate 71% consensus, the experts concurred that IPOM plus is the best option.

**Consensus statement 5:** IPOM plus is the MAS procedure of choice for a primary epigastric hernia of width < 4 cm.

#### Preferred MAS procedure for M5 hernia

The “suprapubic hernia” is defined by the EHS classification as hernia M5 [18]. The uncommon appearance of a suprapubic hernia makes it a significant barrier to the effective treatment of this type of hernia due to the lack of experience in performing open or laparoscopic surgery [30]. Following suprapubic hernia surgery, inadequate mesh overlap brought on by proximity to neurovascular systems leads to high recurrence rates [31]. To obtain a suitable mesh overlap of 5 cm, laparoscopic surgery provides good exposure for identifying the hernia defect as well as neurovascular and bone structures [31]. Hirasa et al. [32] performed laparoscopic treatment for suprapubic hernias in seven patients without dissecting the region of Retzius using a dual-surface mesh with a 2–3 cm overlap and tack fixation. Throughout a mean follow-up time of 5.8 months, they recorded one recurrence [30, 32]. Maemoto et al. [33] described a modified TAPP technique for suprapubic incisional hernia repair which may contribute to decreased recurrence and seroma formation. Varnell et al. [34] studied the morbidity associated with laparoscopic repair of suprapubic hernia and concluded that considering the complexity of the procedure, laparoscopic suprapubic hernia repair is safe and successful with a low recurrence rate of 6.3%.

*Experts’ opinion/consensus recommendation:* The MAS procedure options given to the experts for M5 hernia were IPOM plus, eTEP, TARM, and TAPP. The experts agreed that EP is the preferred choice with a high 100% agreement.

**Consensus statement 6:** EP is the preferred MAS procedure for M5 hernia.

#### Preferred MAS procedure for subxiphoid hernia < 8 cm

A complication of median sternotomy is subxiphoidal incisional hernia, which has a documented incidence of up to 4.2% [35]. According to reports, male sex, obesity, postoperative wound infection, and left ventricular failure are risk factors for subxiphoidal incisional hernia [35]. Owing to its unique anatomic location and the lateral distracting pressures while breathing and coughing, the subxiphoidal hernia repair is quite difficult and prone to recurrence [35, 36]. Various techniques have been developed for the repair of hernias, both open surgical and laparoscopic hernia repair. However, the limited information from retrospective studies can only provide very limited guidance on the best way to repair subxiphoidal hernias [35]. With primary midline approximation of the fascia, conventional hernia repair has subpar results, with recurrence rates as high as 80% [36]. Various open repair methods (onlay mesh, sublay) are discussed for subxiphoid hernia [18].The first report on the laparoscopic repair of a subxiphoid hernia using a bilayer permanent composite mesh and four transmural corner stitches and tacks for fixation to the posterior rectus sheath was published in 2000 [18].

*Experts’ opinion/consensus recommendation:* For subxiphoid hernias < 8 cm, the choices for the MAS procedure were IPOM plus, eTEP, TARM, and TAPP. TAPP achieved 14% consensus, IPOM plus and eTEP achieved 21.5% consensus and TARM achieved 43% consensus.

**Consensus statement 7:** The consensus was not achieved as the experts were unable to come to an agreement on the preferred method for subxiphoid hernias < 8 cm.

#### Preferred procedure for an incisional hernia in vertical single midline incision < 8 cm

The rate of incisional hernia after midline incision is between 2 and 20%, which is commonly underestimated. Incisional hernia is a significant postoperative issue. Higher rates of recurrence make incisional hernia treatment challenging [37]. Despite current guidelines being published and continuous research to find the best closure techniques to prevent incisional hernias, surgeons still frequently encounter incisional hernias [38]. Cochrane review by Brown et al. [39] stated that there was a statistically significant increase in incisional hernias after midline incision compared to transverse incision. Compared to midline incisions, paramedian and transverse incisions have a decreased incidence of incisional hernias [39]. Incisional hernias are frequently treated with open, laparoscopic, and robotic procedures that must be adapted to the patient and hernia’s characteristics [38].

*Experts’ opinion/consensus recommendation:* The options presented to the experts for the preferred procedure for an incisional hernia in the presence of a vertical single midline incision were IP and EP. The experts agreed that IP would be the preferred procedure with a high 79% agreement.

**Consensus statement 8:** In the presence of a vertical single midline incision, IP would be the preferred procedure for an incisional hernia.

#### Preferred MAS approach in a midline incisional hernia of width < 4 cm in a patient with previous laparotomy

In an expert consensus guided by a systematic review, the panel decided that a sublay mesh site is recommended for open elective incisional hernia repair; however, open IPOM may be effective in some circumstances. Mesh procedures for incisional hernias compared to suture techniques resulted in much decreased recurrence rates, as strikingly proved by two published systematic reviews and meta-analyses, registry research, and other studies [40]. But there is still disagreement over which mesh approach will produce the best results for a particular patient. Although meta-analyses have shown the benefits of laparoscopic vs. open treatment of incisional hernias, the laparoscopic IPOM approach is only advised for defects up to 10 cm in size [40].

*Experts’ opinion/consensus recommendation:* The preferred MAS approach options in patients with a prior laparotomy and a midline incisional hernia of width < 4 cm were IPOM plus, eTEP, TARM, and TAPP. A high 93% of the experts concurred that IPOM plus is the best choice.

**Consensus statement 9:** In patients with a previous laparotomy and midline incisional hernia of width < 4 cm, IPOM plus is the preferred MAS approach.

#### Preferred MAS approach in a midline incisional hernia of < 8 cms with multiple defects in a patient with previous laparotomy

According to data from the USA from 2013, 20–27% of the ventral and abdominal incisional hernias were repaired by the laparoscopic technique. A meta-analysis found no difference in recurrence between laparoscopic and open repair for ventral and incisional hernias, although laparoscopic surgery has the advantage of lowering surgical site infection (1.6% vs. 10.1%) [41]. Incisional hernia is a frequent complication of an abdominal surgery. It is reported that prophylactic mesh reinforcement (PMR) uses an onlay or retromuscular approach to perform a midline laparotomy, which results in a considerable decrease in the incidence of incisional hernia in high-risk individuals [42]. As per the SAGES guidelines, it is more helpful to refer to the entire area as a single defect, spanning the full gap between the rectus muscles, in patients who have an incisional hernia from a prior midline incision with multiple hernia defects [17]. In 721 patients with midline incisional hernias, laparoscopic suture closure of the midline defect with mesh reinforcement was described by Palanivelu et al. [43] with an average follow-up of 4.2 years, with a recurrence rate of 0.55% [17, 43].

*Experts’ opinion/consensus recommendation:* The preferred MAS approach options given to the experts for midline incisional hernia with multiple defects in patients with previous laparotomy were IPOM plus, eTEP, TARM, and TAPP. The experts agreed that IPOM plus is the preferred MAS approach with a moderate 64% agreement.

**Consensus statement 10:** In patients with previous laparotomy and midline incisional hernia with multiple defects, IPOM plus is the preferred MAS approach.

### Lateral hernias

#### Preferred MAS procedure for subcostal hernia < 8 cm

Subcostal hernias are “between the costal margin and a horizontal line 3 cm above the umbilicus” [44]. It is a form of lateral abdominal wall hernia that typically develops following open hepatobiliary and esophagogastric procedures [45]. A standard retromuscular technique has typically been used for surgical treatment of a subcostal hernia, with a low recurrence rate. A multicenter Italian study demonstrated that a subcostal hernia could be successfully treated with laparoscopic surgery, avoiding the need for open surgery and preventing the recurrence of the hernia. They also noted that the non-midline VHs with subcostal hernias had the quickest surgical times, which could be attributed to the simplicity of port insertion and adhesiolysis for “localized defect” in the subcostal area. Laparoscopic mesh fixation for a subcostal hernia might be technically difficult, nonetheless, due to the limited surrounding tissue and closeness to bony structures. Different mesh fixation methods for subcostal hernias have been proposed, employing tacks, sutures, adhesives, or a mix of these materials [46].

*Experts’ opinion/consensus recommendation:* IP, EP, and open were the preferred MAS procedure options for subcostal hernias < 8 cm. With a moderate 62% support, the experts decided that EP is the best option.

**Consensus statement 11:** EP is the preferred MAS procedure for subcostal hernia < 8 cm.*Preferred MAS procedure for L3 hernia* < *4 cm.*

The L3 hernia is defined as “iliac (between a horizontal line 3 cm below the umbilicus and the inguinal region)” [44].The extraperitoneal approach is the only method that allows for adequate covering of the visceral sac regardless of the size and location of the defect. Cavalli et al. [47] studied the extraperitoneal approach for complex flank, iliac, and lumbar hernia and concluded that every complex lateral hernia requires the same extensive repair due to the critical anatomy of the region with a big medium weight to heavyweight PPM placed in an extraperitoneal plane.

*Experts’ opinion/consensus recommendation:* The preferred MAS procedure options given to the experts for L3 hernia < 4 cm were IP, EP, and TAPE. The experts agreed that EP is the preferred choice with a high 77% agreement.

**Consensus statement 12:** EP is the preferred MAS procedure for L3 hernia < 4 cm.

### Complications of VHR

#### Occurrence of bowel injuries

The advantages of minimally invasive VHR vs. OVHR include a lower risk of surgical site infections, shorter hospital stays, and a quicker return to normal activities. However, compared to open repairs, laparoscopic repairs had higher enterotomy rates identified by prospective studies and large systematic reviews. Furthermore, Thomas et al*.* [48] examines the effect of a minimally invasive VHR technique on intestinal injury using a nationwide registry of more than 10,000 patients and discovered that LVHR is more likely to result in bowel damage than robotic repair. Henriksen et al. [49] reported that in comparison to open repair, laparoscopic repair resulted in twice as many reoperations for intestinal obstruction or resection.

*Experts’ opinion/consensus recommendation:* Based on the literature review, experts were questioned about the potential for bowel injuries in IP and EP. With a high 77% agreement, the experts opined that there are comparable chances of bowel injuries in EP and IP repairs.

**Consensus statement 13:** There are equivalent chances of bowel injuries in EP hernia repairs compared to IP repairs.

#### Occurrence of potential neurovascular injuries

Depending on the repair technique, the incidence of neuralgias ranges from 0.5 to 4.6%. The IPOM technique was dropped as a feasible repair option because it had the greatest incidence of neuralgias in one study. The “genitofemoral nerve, the intermediate cutaneous nerve of the thigh, and the lateral cutaneous nerve of the thigh” are the most often affected nerves [49].

*Experts’ opinion/consensus recommendation:* The question of whether neurovascular injuries occur more frequently in IP or EP was put to the experts. There was a unanimous consensus among the specialists that potential neurovascular injuries are more likely in EP.

**Consensus statement 14:** Potential neurovascular injuries are more common in EP.

#### Occurrence of hematomas/vascular injuries

The incidence of hematoma has been reported to be 3%–8% after TAPP. According to the published meta-analysis, the average incidence is 3–4%. Chronic anticoagulation, recurrent hernia surgery, mesh fixation, a bigger hernia defect, and medial defect localization enhance the risk of hematoma. Hematomas frequently appear soon after the operation, and imaging tests reveal a fluid accumulation [50]. Vascular injuries are among the most frequent injuries encountered after hernia repair and are frequently the cause of conversion. It can happen at several different locations, including the “rectus muscle vessel during trocar insertion, the inferior epigastric vessel, bleeding from the pubic symphysis venous plexus, an aberrant obturator vein injury, testicular vessel injury, and the most dangerous of all, the iliac vessels”, which require an emergency conversion to stop the bleeding and the prompt assistance of a vascular surgeon to be repaired [49].

*Experts’ opinion/consensus recommendation:* Experts were asked to give their opinion on whether hematomas/vascular injuries are more common in IP than in EP. The experts opined that hematomas/vascular injuries are more common in EP with a high 100% agreement.

**Consensus statement 15:** Hematomas/vascular injuries are more common in EP.

#### In-hospital pain after IPOM plus with TAP block

Following VHR in the IPOM technique, patients frequently have postoperative acute and chronic pain [51]. Nguyen et al*.* [52] studied the postoperative pain after laparoscopic VHR in comparison of sutures vs. tacks and found no difference seen in postoperative pain in patients undergoing laparoscopic VHR with primarily transabdominal sutures or tacks. Recent research has examined the effectiveness of transversus abdominis plane (TAP) block in hernia repair to reduce acute and chronic pain following surgery and expedite early recovery. Meyer et al. [53] reported that TEP with TAP block inguinal hernia without curare is effective, safe, and reproducible and can be proposed in all patients. Feierman et al. [54] observed that the TAP block had a sufficient analgesic effect for 13 patients who had open umbilical hernia surgery. Additionally, a prospective RCT with 70 patients who had VHR demonstrated that a TAP block could reduce pain [51, 54]. Paasch et al. [55] reported that in some patients, the TAP block performed before laparoscopic VH surgery may lessen early postoperative pain and analgesic prescription use.

*Experts’ opinion/consensus recommendation:* On whether in-hospital discomfort following IPOM plus with TAP block is comparable to EP repairs, a high consensus was achieved with 77% of experts agreeing that hospital pain associated with IPOM plus with TAP block is comparable to that of EP repairs.

**Consensus statement 16:** The in-hospital pain after IPOM plus with TAP block is equivalent to that of EP repairs.

#### Adhesions occur in IPOM with a coated mesh

More than 50% of abdominal surgeries result in intra-abdominal adhesions, which are a primary cause of postoperative problems. Mesh grafts with antiadhesive properties relative to the viscera have been introduced [56]. Chowbey et al. [57] reported about 202 laparoscopic VHR using pure PPMs and did not note any bowel adhesions [57, 58]. Up to two-thirds of laparoscopic VHR are reportedly being done in low- and middle-income countries with unprotected PPM due to cost [58].The intestinal adhesion to mesh and consequent complications have been a prominent issue with IPOM. However, the initial animal experiment and more current research have demonstrated that the actual incidence is significantly lower than thought, especially with the new-generation coated meshes [59]. No problems due to intestine adhesion to mesh were discovered when composite collagen-coated PPM was used during laparoscopic IPOM repair for primary inguinal hernia [59].

*Experts’ opinion/consensus recommendation:* Based on the literature review, experts were asked whether adhesions occur in IPOM with a coated mesh. Experts agreed that clinically insignificant adhesions occur in IPOM with a coated mesh with a high 92% agreement.

**Consensus statement 17:** Clinically insignificant adhesions occur in IPOM with a coated mesh.

#### Preferred approach in a patient with symptomatic hernia, BMI > 40 kg/m^2^

In an algorithm that was published in 2020, the recommendation suggested if there is an abdominal or an incisional hernia, for symptomatic cases and all defect sizes, a hernia repair should be followed by bariatric surgery. If the hernia is < 4 cm (W1) and asymptomatic, then concomitant bariatric surgery should be done with hernia repair. In asymptomatic cases with defect > 4 cm (W2–W3), bariatric surgery should be done with subsequent laparoscopic VHR. However, this study did not factor in the BMI of patients, it only factored in the size of the defect [60]. Baig et al. [61] published their work on patients with a BMI of 30 kg/m^2^. The study factored in the BMI and the size of the defect accordingly.

Asymptomatic cases:

- With obesity (BMI < 40 kg/m^2^) and defect size < 8 cm, IPOM plus is recommended after weight loss or Bariatric surgery.
- With obesity (BMI < 40 kg/m^2^) and defect size > 8 cm, an abdominal wall reconstruction procedure is recommended after weight loss or Bariatric surgery.
- With obesity (BMI > 40 kg/m^2^) and defect size < 8 cm, Bariatric surgery followed by IPOM plus are recommended.
- With obesity (BMI > 40 kg/m^2^) and defect size > 8 cm, Bariatric surgery followed by Abdominal wall reconstruction are recommended.

*Experts’ opinion/consensus recommendation:* In patients with symptomatic hernia, BMI > 40 kg/m^2^, and defect up to 4 cm, IP and EP were the surgical options that experts voted on. Experts agreed that IP is the preferred approach with a high 100% agreement.

**Consensus statement 18:** IP is the preferred approach in patients with symptomatic hernia, BMI > 40 kg/m^2^, and defect up to 4 cm.

#### Preferred approach in patients indicated for MAS VH surgery having grade 3/4 ASA

Based on demographic variables [age, sex, BMI, American Society of Anaesthesiologists (ASA), presence of preoperative sepsis, wound classification, and comorbidities], 1642 patients receiving emergency care for primary or incisional VH (with bowel obstruction or gangrene) were balanced into two groups (laparoscopic and open) using a propensity score-matched method. In the laparoscopic group, the mortality rate was 1.3%, whereas in the open group, it was 1.1% [relative ratio (RR), 1.22; 95% confidence interval (CI), 0.51–2.93]. In the univariable analysis, the laparoscopic approach showed an overall morbidity rate of 9.1% vs. 15.1% of the open approach (RR, 0.60; 95% CI 0.46–0.79). Abdominal wall complications (superficial and deep wound infections, and dehiscence) were 3.0% vs. 7.9% (RR, 0.38; 95% CI 0.24–0.60) [62].

*Experts’ opinion/consensus recommendation:* In patients indicated for MAS VH surgery having grade 3/4 ASA, IP and EP were the options given to the experts. With a high 93% consensus, experts felt that IP is the best strategy.

**Consensus statement 19:** IP is the preferred approach in patients indicated for MAS VH surgery having grade 3/4 ASA.

### Factors that influence procedural selection of VHR

The purpose of VHR is to alleviate symptoms and/or stop further hernia-related issues, including discomfort, acute incarceration, expansion, and skin issues [16]. All hernia repairs require defining the surgical goals preoperatively between the patients and the surgeon [16]. When considering a laparoscopic technique, it is vital to take the hernia defect size into account because larger defects typically make the treatment more challenging [16]. Some VHs are too large to approximate the fascia without using advanced methods. Large VHs are defects with a width greater than 10 cm as defined by the European Hernia Society. However, several parameters, such as weight of the patient, BMI, abdominal wall compliance, and hernia location (e.g., subxiphoid, periumbilical, and suprapubic), affect the width at which the fascia can no longer be reapproximated without advanced procedures. The surgeon should think about the patient’s comorbidities, the setting (emergency or elective), the size of the defect, the quantity of release that would be necessary, the level of contamination, the situation (emergency or elective), and the hospital’s capacity to care for this patient while preparing a surgical approach [63].

*Experts’ opinion/consensus recommendation:* Factors that dictate the choice of a particular procedure with options of width, location, and BMI were asked of the experts. Experts agreed that the width, location, and BMI were all to be considered with a high 100% agreement for width and location and 93% agreement for BMI.

**Consensus statement 20:** Width, location, and BMI are the factors that dictate the choice of a particular procedure.

### Algorithm for procedural selection of VHR

#### Algorithm for the selection of procedures for VH based on hernia defect size (Table 2)

**Table 2:** Algorithm for procedure selection in VH [61, 64]

Hernia defect size	Procedural selection
For < 2 cm, primary, asymptomatic cases	Suture repair/IPOM
For < 2 cm, recurrent/incisional hernias	IPOM is recommended, IPOM plus/open sublay
2–4 cm and 2–6 cm in obese	IPOM plus
With 5–8 cm/divarication	eTEP–RS
For 9–12 cm with overlying normal skin, atypical location, and recurrent cases	Component separation/open endoscopic/TARM/eTEP–TAR/TAPP
With defects > 12 cm	Open TAR
For 9–12 cm with redundant skin; any width with adverse wound factors	Open RS/ACST/PCST/onlay myocutaneous flaps
Asymptomatic BMI < 40 kg/m^2^ Defect size < 8 cm Defect size > 8 cm	Weight loss/ Bariatric Surgery followed by IPOM plus Weight loss/ Bariatric surgery followed by Abdominal wall reconstruction (AWR)
Asymptomatic BMI > 40 kg/m^2^ Defect size < 8 cm Defect size > 8 cm	Bariatric surgery followed by/IPOM plus Bariatric Surgery followed by AWR

Dr. Igor Belyansky invented and popularized new techniques and nomenclatures including Extended Totally Extraperitoneal Rives-Stoppa (eTEP-RS) and Extended Totally Extraperitoneal Transversus Abdominis Release (eTEP-TAR), which involved posterior component separation with limited access. Baig et al. [61] and Baig et al. [64] developed an algorithm based on the data from the literature and attempted to include these treatments into their hernia surgery. Hernia width was a key factor in their procedure selection [61, 64].

Based on the recommendations from the adboard, the consensus statements were finalized and the algorithm was developed (Table 3) to support and enhance the decision-making for VHR.

**Table 3:** An algorithm based on the present consensus statements

Nature of hernia defect	Preferred procedure
Valid options for MAS in VH	IPOM plus/TARM/eTEP/MILOS/TAPP/TAPE/SCOLA/LIRA
Valid options for hernia defect ≤ 8 cm	IPOM plus/TARM/eTEP/MILOS/TAPP/TAPE/SCOLA/LIRA
Preferred MAS procedure for primary umbilical hernia < 4 cm without diastasis	IP
Valid MAS procedure of choice for a primary epigastric hernia of width < 4 cm	IPOM plus
Preferred MAS procedure for M5 hernia	EP
An incisional hernia in vertical single midline incision	IP
MAS approach in a midline incisional hernia of width < 4 cm in a patient with previous laparotomy	IPOM plus
Midline incisional hernia with multiple defects in a patient with previous laparotomy	IPOM plus
Subcostal hernia < 8 cm	EP
L3 hernia < 4 cm	EP
Symptomatic hernia, BMI > 40 kg/m^2^, and defect up to 4 cm	IP
MAS VH surgery having grade 3/4 ASA	IP
Factors that dictate the choice for a particular procedure	Width, location, and BMI

## Discussion

The adoption of laparoscopic procedures for VHR is increasing with an increasing interest in laparoscopic surgery and the availability of new materials. The best cosmetic outcomes, a quicker recovery time, and minimum postoperative pain are the key benefits of LVHR over the open technique, according to the current literature [65]. A study by Luque et al. [66] reported that with the exception of seroma rate, on the 1st, 7th, and 31st postoperative days, the eTEP group had less discomfort than the IPOM plus group (p < 0.05); improved functional recovery on the 30th and 180th day; and improved cosmetic appearance than IPOM plus. Basukala et al. [67] compared outcomes after IPOM vs. IPOM plus for VH and found that even though the procedure takes longer and the hospital stay is longer, the probabilities of a six-month recurrence following the IPOM procedure were 14.86 (95% CI 2.51–87.85, p = 0.003) times greater than those following IPOM plus the type of repair when the confounding variables were adjusted. Similar findings have been reported by another study, which found that VHR using laparoscopic IPOM plus is a safe, highly effective, and widely used procedure with a very low recurrence rate (0.4%) [68].

While inguinal hernia is well-researched, there is a gap in the research for ventral/incisional hernias. Among the hernias presented in patients nowadays, many are abdominal incisional hernias. Currently, there are many VHR surgical techniques, but the classification and degree of staging of hernia are lacking in the literature. Such staging would help assess the right surgical approach and avoid complications. A hernia is a benign condition, and the surgery is a repair approach, aiming to improve the patient’s quality of life. Thus, standardizing the practice of laparoscopic VHR is the need of the hour. There is a lack of high-level evidence in support of the different types of VHR techniques, which emphasizes the need for a consensus. This consensus document will give guidance on the decision-making process for laparoscopic VHR and standardize the practice of laparoscopic VHR with a focus on IPOM repair.

## Conclusion

This article summarizes the Delphi consensus on MAS approaches for VHR in the Indian scenario. Experts recommended IPOM plus/TARM/eTEP/MILOS/TAPP/TAPE/SCOLA/LIRA as valid MAS options for VH and are usually feasible for detecting widths up to a maximum of 8 cm to enable tension-free closure. IP is the preferred MAS procedure for primary umbilical hernia < 4 cm without diastasis; incisional hernia in the presence of a vertical single midline incision; in patients with a symptomatic hernia, BMI > 40 kg/m^2^, and defect up to 4 cm; and in patients indicated for MAS VH surgery having grade 3/4 ASA. IPOM plus is the preferred MAS procedure for midline incisional hernia of width < 4 cm in patients with a previous laparotomy; midline incisional hernia with multiple defects in patients with a previous laparotomy; and primary epigastric hernia of width < 4 cm. EP is the preferred MAS procedure for L3 hernia < 4 cm, midline hernias < 4cms with diastasis, subcostal hernia < 8 cm, and M5 hernia.

## Data Availability

The data used to support the findings of this study are included within the article.
